# *MLLT1* YEATS domain mutations in clinically distinctive Favourable Histology Wilms tumours

**DOI:** 10.1038/ncomms10013

**Published:** 2015-12-04

**Authors:** Elizabeth J. Perlman, Samantha Gadd, Stefan T. Arold, Anand Radhakrishnan, Daniela S. Gerhard, Lawrence Jennings, Vicki Huff, Jaime M. Guidry Auvil, Tanja M. Davidsen, Jeffrey S. Dome, Daoud Meerzaman, Chih Hao Hsu, Cu Nguyen, James Anderson, Yussanne Ma, Andrew J. Mungall, Richard A. Moore, Marco A. Marra, Charles G. Mullighan, Jing Ma, David A. Wheeler, Oliver A. Hampton, Julie M. Gastier-Foster, Nicole Ross, Malcolm A. Smith

**Affiliations:** 1 Department of Pathology, Ann & Robert H. Lurie Children's Hospital of Chicago, Northwestern University's Feinberg School of Medicine, 225 E. Chicago Ave, Chicago, Illinosis 60611, USA; 2 King Abdullah University of Science and Technology, Department of Biochemistry and Molecular Biology, Division of Biological and Environmental Sciences and Engineering, Computational Bioscience Research Center, Thuwal 23955, Saudi Arabia; 3 Office of Cancer Genomics, National Cancer Institute, 31 Center Drive, Bethesda, Maryland 20892, USA; 4 Department of Genetics, The University of Texas MD Anderson Cancer Center, 1515 Holcombe Blvd., Houston, Texas 77030, USA; 5 Department of Pediatrics, Division of Pediatric Hematology/Oncology, Children's National Medical Center, 111 Michigan Avenue, NW, Washington DC 20010, USA; 6 Center for Biomedical Informatics and Information Technology, National Cancer Institute, National Institutes of Health, 9609 Medical Center Drive, Bethesda, Maryland 20892, USA; 7 Frontier Science and Technology Research Foundation, 505 S. Rosa Rd #100, Madison, Wisconsin 53719, USA; 8 Canada's Michael Smith Genome Sciences Centre, British Columbia Cancer Agency, Vancouver, British Columbia, Canada V5Z 4S6; 9 Department of Pathology, St Jude Children's Research Hospital, 262 Danny Thomas Place, Mail Stop 342, Memphis, Tennessee 38105, USA; 10 Department of Pathology and Laboratory Medicine, Nationwide Children's Hospital, Ohio State University College of Medicine, Columbus, Ohio 43205, USA; 11 Department of Molecular and Human Genetics, Baylor College of Medicine, Houston, Texas 77030, USA; 12 Departments of Pathology and Pediatrics, Ohio State University College of Medicine, 700 Children's Drive, Columbus, Ohio 43205, USA; 13 Cancer Therapy Evaluation Program, National Cancer Institute, 9609 Medical Center Drive, RM 5-W414, MSC 9737, Bethesda, Maryland 20892, USA

## Abstract

Wilms tumour is an embryonal tumour of childhood that closely resembles the developing kidney. Genomic changes responsible for the development of the majority of Wilms tumours remain largely unknown. Here we identify recurrent mutations within Wilms tumours that involve the highly conserved YEATS domain of *MLLT1* (ENL), a gene known to be involved in transcriptional elongation during early development. The mutant MLLT1 protein shows altered binding to acetylated histone tails. Moreover, *MLLT1*-mutant tumours show an increase in *MYC* gene expression and HOX dysregulation. Patients with *MLLT1*-mutant tumours present at a younger age and have a high prevalence of precursor intralobar nephrogenic rests. These data support a model whereby activating *MLLT1* mutations early in renal development result in the development of Wilms tumour.

## Abstract (web summary)

Wilms tumour is a rare renal neoplasm that primarily affects children but the genomic changes responsible for its development are currently largely unknown. In this study, the authors identify somatic mutations of the *MLLT1* gene that are potentially involved in the aetiology of a subset of Wilms tumours.

## Graphical abstract

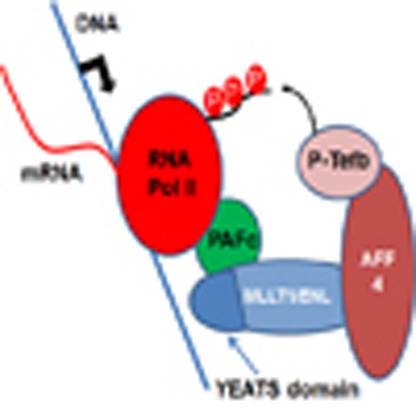

Wilms tumour (WT), the most common embryonal neoplasm of the childhood kidney, has long intrigued investigators by its histologic similarity to the developing kidney^1^. WTs are also noteworthy for their associated precursor lesions known as perilobar and intralobar nephrogenic rests (PLNR, ILNR)^2^. Favourable histology Wilms tumours (FHWT) represent 95% of WT; the remaining 5% develop an additional clonal event (most commonly *TP53* mutation) resulting in unfavourable histology characterized as anaplasia (nuclear hyperchromasia and pleomorphism with polyploid mitoses)^3^,^4^. Molecular alterations long known to contribute to WT development include mutations and/or deletions involving *WT1*, *WTX*, *CTNNB1* and loss of imprinting or loss of heterozygosity (LOH) at 11p15 (reviewed in ref. 5). However, the genomic changes responsible for the development of most WTs remain unknown. The National Cancer Institute's ‘Therapeutically Applicable Research to Generate Effective Treatments' (TARGET) initiative seeks to identify therapeutic targets for high-risk paediatric tumours through comprehensive characterization and integrative analyses (http://ocg.cancer.gov/programs/target). The ongoing High Risk TARGET Wilms Tumours include two groups: FHWT that subsequently relapsed and anaplastic WT.

We report in-frame insertion/deletion *MLLT1* mutations in FHWT that are absent in other TARGET paediatric tumour types. These *MLLT1* mutations were associated with changes in binding to H3K9ac, evidence of *HOX* and *MYC (MYC-C)* expression dysregulation, and were often accompanied by *CTNNB1* mutations and evidence of Wnt pathway activation. Further, patients with *MLLT1* mutations presented at a younger age and were associated with ILNR rather than PLNR. We conclude that activating *MLLT1* mutations accompanied by Wnt activation early in renal development result in the development of Wilms tumour.

## Results

### Identification and characterization of *MLLT1* variants

**Figure 1:**
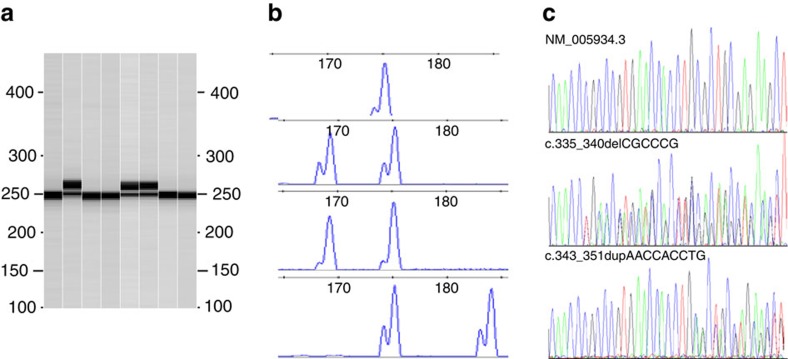
*MLLT1* variant verification. (**a**) Base-pairs (242) of genomic DNA were amplified and the product was detected by capillary electrophoresis. Eight representative samples with three containing additional larger bands identifying insertion mutations (lanes 2, 5 and 6) are shown. Samples with novel bands were confirmed by Sanger sequencing. (**b**) The relative expression of the mutant and wild-type alleles was determined using RT–PCR and primers designed to amplify exons 4 and 5 of the *MLLT1* gene (NM_ 005934) with detection by capillary electrophoresis. The normal allele is represented by a peak at ∼175 bp (top panel). Two samples show expression of a shorter allele corresponding to deletion mutations (second and third panels) and one sample shows expression of a longer allele (bottom panel) corresponding to an insertion mutation. It should be noted that the mutant alleles are expressed at approximately the same level as the normal alleles. (**c**) Samples with abnormal bands identified following PCR amplification were confirmed by Sanger dideoxy sequencing. Shown are three samples including the reference sequence on top (NM_005934.3), a 6-nucleotide deletion in the middle (c.335_340delCGCCCG) and a 9-nucleotide duplication on the bottom (c.343_351dupAACCACCTG). The base-pair positions have been aligned to show that these affect the same domain.

As we recently reported, analysis of 77 FHWT by whole genome sequencing (WGS, 58 patients) or whole exomic sequencing (WES,19 patients) identified 825 high-quality somatic, non-synonymous variants, with an average of 11 candidate mutations/case (range 2–42) (ref. 6). Six variants (all identified by WGS) involved *MLLT1*, a gene not previously reported to be involved in WT. Each mutation was verified by Sanger sequencing of PCR products spanning the variants (Fig. 1). Other verified mutations identified include somatic single nucleotide variants (SNVs) or small deletions in *WT1* (three patients, 4.5%), *CTNNB1* (five patients, 6.5%), *WTX* (also known as *FAM123B* or *AMER1*, five patients, 6.5%), *DROSHA* (seven patients, 10%), *DGCR8* (three patients, 4.5%), and *SIX1* or *SIX2* (eight tumours, 10%)^6^. Two tumours with *MLLT1* mutations also had *CTNNB1* mutations; no *MLLT1*-mutant tumours had accompanying *WT1*, *WTX*, *DROSHA*, *DGCR8*, *SIX1*, or *SIX2* mutations (Supplementary Table 1). These represent all genes with verified variants in at least four patients with FHWT (>5%); the full TARGET high-risk Wilms tumour data, including 125 tumours (both favourable histology and anaplastic tumours) and including verified variants present at lower frequencies, will be reported in full when these data become available.

Four cases had the identical nine nucleotide in-frame insertion (AACCACCTG) at *MLLT1* position c.343_351 (p.117_118insNHL) of the transcript NM_005934.3. Two mutations were nearby in-frame deletions, one with deletion of six nucleotides at position c.335_340 (p.112_114PPV>L) and one with a seven-nucleotide deletion and a one-nucleotide insertion at position c.333_339 (p.111_113NPP>K) (Supplementary Table 1). The *MLLT1* mutations were present in 49–73% of the sequencing reads and reverse transcription PCR (RT–PCR) analysis found expression of both wild-type and mutant alleles. Similar *MLLT1* insertion/deletion variants were not identified in other TARGET tumours (neuroblastoma, osteosarcoma, acute lymphocytic and myelogenous leukaemia, unpublished), nor have they been reported in dbSNP 134 or 135 (ref. 7), COSMIC v69 (ref. 8), or in the 1000 Genomes Pilot Projects 1, 2, and 3 (ref. 9). The PROVEAN algorithm^10^ predicted these *MLLT1* mutations to be deleterious (scores <−10). PCR amplification followed by sequencing identified an additional *MLLT1* p.117_118insNHL mutation in 1/19 tumours originally characterized by WES, for a total of 7 *MLLT1* mutations in 77 discovery set tumours (11%) (Supplementary Table 1). No germline *MLLT1* mutations were identified.

### Copy number and gene expression analysis

**Figure 2:**
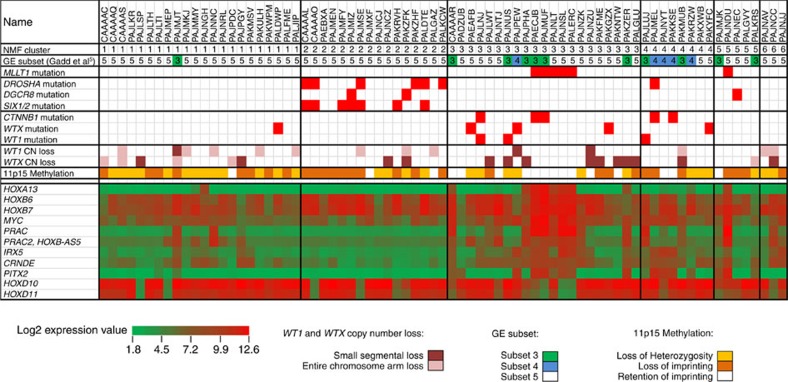
Unsupervized analysis of gene expression data. Non-negative Matrix Factorization (NMF) analysis of 75 FHWT resulted in 6 clusters with the highest cophenetic correlation (0.95) after *k*=2. Five of six *MLLT1* mutant tumours with available gene expression data occurred in NMF cluster 3, and two were accompanied by *CTNNB1* mutations. This cluster also contained four tumours with mutation or small segment deletion of *WT1*, all of which also had either a mutation of *CTNNB1* or small segment deletion or mutation of *WTX*. This cluster also contained a substantial number of tumours with retention of imprinting of 11p15 (including all *MLLT*1-mutant tumours). The sequencing and copy number data is provided in Supplementary Table 1. The predicted membership in gene expression subsets previously reported^5^ is provided. (Subsets 1 and 2 were low risk tumours and are therefore not represented in TARGET, Subsets 3 and 4 are highlighted in green and blue, respectively, and Subset 5 in white). Illustrated at the bottom are the expression patterns of genes of interest that were highly significantly differentially expressed between *MLLT1*-mutant and wild-type tumours.

**Figure 3:**
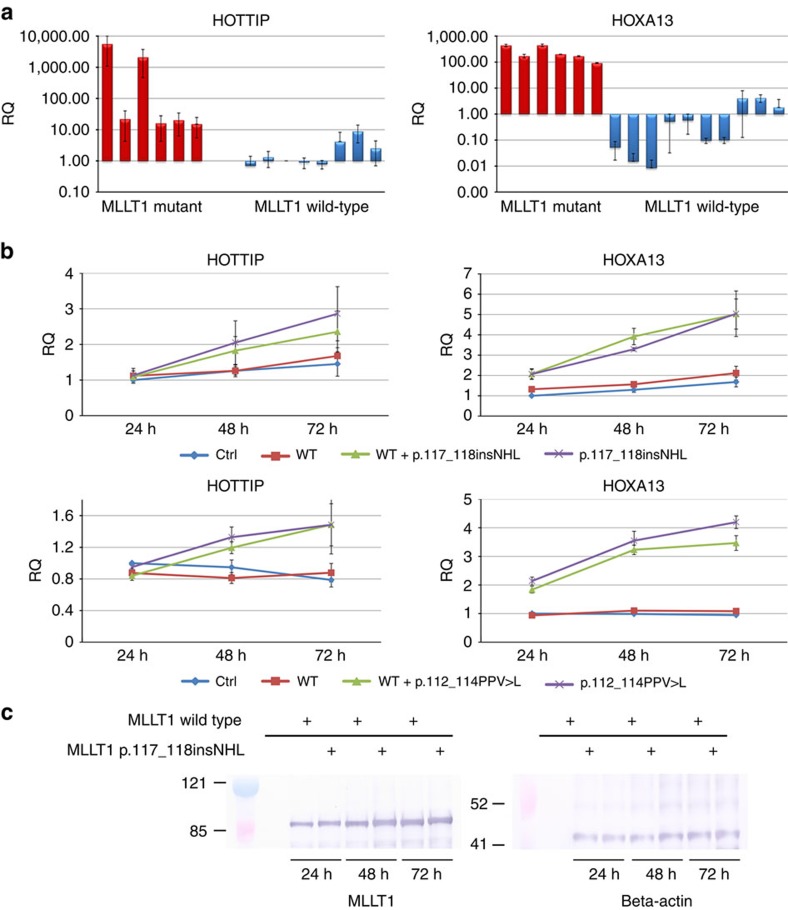
Quantitative RT–PCR for *HOTTIP* and *HOXA1*3. (**a**) FHWT samples with *MLLT1* mutations (*n*=6; red) and randomly selected *MLLT1*-wild-type FHWT (*n*=10; blue) were evaluated for *HOTTIP* and *HOXA13* expression levels by RT–PCR normalized to endogenous GAPDH levels. A FHWT lacking *MLLT1* mutation was used as the reference sample for calculating the relative quantitation (RQ) value. Samples were run in duplicate and the error bars represent the s.e.m. of the RQ values. The data are presented in log scale. Note that for tumours that lack expression a bar is not visible. (**b**) HEK293 cells transfected with empty vector (Ctl), wild-type *MLLT1* (WT), a 1:1 ratio of wild-type *MLLT1* and either p.117_118insNHL (upper panels) or p.112_114PPV>K (lower panels), or only mutant p117_118insNHL or p.112_114PPV>K is shown. RNA was isolated 24, 48 and 72 h after transfection, and the expression levels of *HOXA13* and *HOTTIP* were normalized to endogenous *GAPDH* levels. The ddCt value for the control sample at 24 h was used as the reference sample for calculating the RQ value in each sample for each gene. Three independent experiments were performed for each transfection and the error bars represent the s.e.m. of the RQ values. (**c**) HEK293 cells transfected with wild-type *MLLT1* or mutant *MLLT1* (p117_118insNHL). Lysates were collected at 24, 48, and 72 h for western blotting with a MLLT1 (left) or beta-actin (antibody). The mutant protein was not significantly differentially expressed compared with the wild-type protein at any of the time points evaluated. Results for p.112_114PPV>K likewise demonstrated no significant difference in levels of protein expression.

Copy number loss at 19p13 (the chromosomal location of *MLLT1*) was not identified in any of the 77 FHWT; five non-mutant tumours had gain of 19p13 due to gain of all or the majority of chromosome 19. Segmental copy number loss was identified for *WT1* (14 tumours, three showing loss of small segments containing all or part of *WT1* and the remainder loss of the majority or all of chromosome 11) and *WTX* (19 tumours, 14 showing loss of small segments containing all or part of *WTX* and the remainder loss of the majority or all of the X chromosome) (Fig. 2, Supplementary Table 1). Gene expression analysis was available for 75 of the 77 tumours (one *MLLT1*-mutant tumour and one *MLLT1* wild-type tumour did not pass quality control). As we recently reported within the same 75 tumours, unsupervized analysis using non-negative matrix factorization (NMF) resulted in *k*=6 clusters having the highest cophenetic correlation (0.95) after *k*=2 (ref. 6). We are now able to identify five of six *MLLT1*-mutant tumours within NMF cluster 3 (Fig. 2). Comparison of global gene expression of *MLLT1*-mutant tumours with 69 *MLLT1* wild-type tumours yielded 96 genes significantly differentially expressed (two-class unpaired significance analysis of microarrays *q*<0.05, Bonferroni-corrected Student's *t*-test *p* value <0.05, fold change >2, Supplementary Table 2). Noteworthy was the significantly increased expression of *HOXA13*, *HOXB6*, *HOXB7*, *MYC*, *PRAC*, *IRX3* and *PITX2*, and decreased expression of *HOXD10* and *HOXD11* (Fig. 2). Of particular interest was the high expression of *HOXA13* (fold change 128), a gene not expressed in the remaining FHWT nor in the cap mesenchyme of the developing kidney^11^. The expression of *HOXA13* mRNA has been shown to require the expression of the immediately adjacent lncRNA *HOTTIP* (refs 12, 13). Six *MLLT1*-mutant tumours and six randomly selected *MLLT1*-wild-type FHWTs were analysed by quantitative reverse transcription PCR, which confirmed increased expression of both *HOXA13* and *HOTTIP* in *MLLT1*-mutant tumours (Fig. 3a). Increased expression of other lncRNA/target pairs, including *PRAC* and its adjacent lncRNA *HOXB-AS* (*PRAC2*, *NCRNA00253*) (ref. 14) and *IRX5* and its adjacent lncRNA *CRNDE* (ref. 15) was also observed (Supplementary Table 2, Fig. 2). The increased expression of these critical lncRNAs detected with array analysis is particularly noteworthy because lncRNAs are not usually expressed at levels detectable using these methods.

To validate the functional impact of the *MLLT1* mutations on gene expression, we transfected the *MLLT1* p.117_118insNHL and p.112_114PPV>K mutant constructs alone or at a 1:1 ratio with the wild-type *MLLT1* construct into the embryonic kidney cell line HEK293. Both *HOXA13* and *HOTTIP* were significantly upregulated in the cells transfected with either of the mutant *MLLT1* genes alone and in the cells transfected with the 1:1 ratio of mutant:wild-type *MLLT1* (Fig. 3b) compared with the vector transfected control cells and the cells transfected with wildtype alone. To confirm that the *MLLT1* mutations did not alter protein stability, cell lysates from the transfected cells were evaluated by western analysis at 24 48, and 72 h (Fig. 3c and Supplementary Fig. 2), revealing no significant differences in mutant protein expression levels.

### *MLLT1* mutation clinical and genetic correlations

The TARGET FHWT cases were selected as high risk on the basis of their clinical recurrence. To determine the frequency of *MLLT1* mutations overall in FHWT, we screened a validation set of 475 FHWT by PCR amplification and sequencing. *MLLT1* insertion or deletion mutations were found in 19/475 tumours (4%). The p.117_118insNHL mutation was identified in 13/19 tumours. Three identical new variants were also identified as p.117_118insHHL. The remaining three were deletion mutations at p.111–112 (Table 1). *MLLT1* mutations were observed in 6% (9/139) of FHWT that recurred, compared with 3% (10/336) of FHWT that did not recur. Although this suggests an association between *MLLT1* mutation and relapse, this difference does not meet statistical significance. There was also no detectable correlation between the specific *MLLT1* mutation and relapse.

The clinical, pathologic and molecular details of the 19 FHWT carrying somatic *MLLT1* mutations compared with the entire group of 475 FHWT are provided in Table 1. The median age at diagnosis of the patients with and without *MLLT1* mutations was 12 and 44 months, respectively, (*P*<0.0001, Wilcoxon test). There was also a significant correlation between *MLLT1* mutation status and nephrogenic rest status. Of MLLT1-wild-type tumours, 23 and 21% were associated with ILNRs and PLNRs, respectively; in contrast, 10/19 (53%, *P*=0.011) MLLT1-mutant tumours arose in ILNRs, and 0/19 (*P*=0.019) arose in PLNRs (Fisher's exact test). Four MLLT1-mutant tumours arose in kidneys with multiple ILNRs, a feature thus far predominantly recognized in patients with syndromes associated with germline *WT1* mutations. Of note, in 5/7 MLLT1-mutant tumours in the discovery set, the comparator constitutional sample was adjacent normal kidney and lacked the mutation. A source of constitutional DNA was available for the four cases in the validation set that had multiple ILNRs (two from peripheral blood and two from adjacent normal kidney) and these samples likewise lacked *MLLT1* variants by PCR amplification and sequencing. Sufficient DNA was available on 16/19 MLLT1-mutant tumours to perform mutation analysis for *WT1*, *CTNNB1*, *WTX*, *DROSHA*, *DGCR8*, and *SIX1/2* and this revealed five *CTNNB1* mutations, one *WTX* mutation, and no *WT1 DROSHA*, *DGCR8*, or *SIX1/2* mutations.

A different prevalence of other genetic markers was identified in MLLT1-mutant tumours. Overall, 81% of all FHWT have either LOH or loss of imprinting at ICR1 on 11p15 as determined by methylation analysis^5^; in comparison, only 2/18 (11%) FHWT with *MLLT1* mutations with available material demonstrated such changes (Table 1). LOH of 1p and 16q are known to be associated with relapse in patients with FHWT^16^. Information regarding 1p and 16q LOH analysis performed prospectively during the protocol was available for 11/19 MLLT1-mutant tumours, and neither was found. Similarly, 1q gain is present in 57 and 24% of patients with FHWT that relapsed and do not relapse, respectively^17^. 1q gain was identified in 1/15 *MLLT1*-mutant FHWT (see methods). Thus, although *MLLT1* mutation was often associated with poor outcome, this association was independent of other prognostic markers.

### MLLT1 protein evaluation

**Figure 4:**
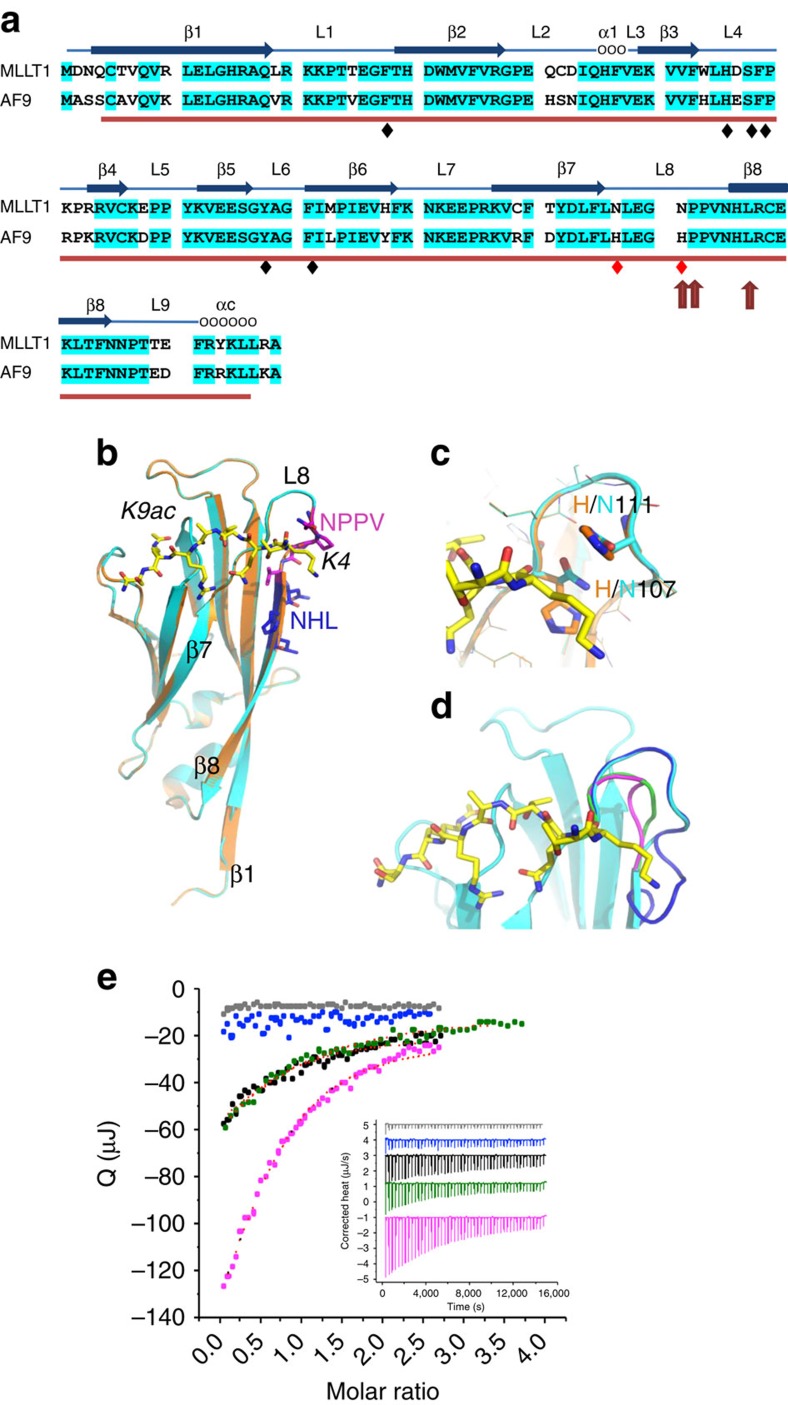
MLLT1 computational sequence and structure analysis. (**a**) The sequences of the MLLT1 and AF9 YEATS domains are provided. Residues forming the YEATS domain are underlined in red. Identical residues are highlighted in blue. The secondary structural elements, including the β strands and intervening loops described for AF9 (ref. 19) are provided. The recognition sites for H3K9ac by AF9 are indicated with a black diamond; residue differences at positions 107 and 111 that are also recognition sites for H3K4 by AF9 are indicated with a red diamond^19^. Arrows indicate the *MLLT1* mutations identified in the current study. (**b**) Homology model of the MLLT1 YEATS domain (cyan), superimposed on AF9 YEATS (orange) bound to H3K9ac (yellow stick model; PDB 4TMP). The motifs in which the indels occur are shown in blue and magenta letters. The β1, β7, β8 strands and L8 loop are designated (see text). (**c**) Amino acid changes between AF9 and MLLT1 in the L8 loop affect the recognition of the N-terminal region of acetylated lysine motifs. Residue differences (H>N) between AF9 and MLLT1 at positions 107 and 111 are highlighted in the superimposition of the AF9 crystal structure (PDB 4TMP; orange) with our homology model of MLLT1 YEATS (magenta). (**d**) The *MLLT1* mutations are predicted to specifically affect the L8 loop. Computational homology models are coloured cyan (wild-type MLLT1), magenta (p.111_113NPP>K), green (p.112_114PPV>L) and blue (p.117_118insNHL). p.117_118insHHL is predicted to produce the same conformation and phenotype as p.117_118insNHL, because N/H117 are solvent exposed at the back of β8. (**e**) Effect of *MLLT1* mutations on binding H3K9ac, measured by ITC, performed in triplicate. Shown are integrated heats resulting from binding of H3K9ac to MLLT1 wild-type (black), p.111_113NPP>K (magenta), p.112_114PPV>L (green) and p.117_118insNHL (blue). Heats resulting from peptide being titrated into buffer are in grey. Inlay: ITC data adjusted to baseline and offset for better visibility.

The *MLLT1* locus encodes a 559aa protein from 12 exons and is one of over 100 proteins that contain a YEATS domain. The domain name is derived from the five proteins first discovered in either *Saccharomyces cereviseiae* or humans, including Yaf9 (YEATS4, GAS41), ENL (MLLT1, YEATS1), AF9 (MLLT3, YEATS3), Taf14 and Sas5. In humans, MLLT1 and AF9 are the most studied and are highly homologous^18^. Using a variety of structural and functional analyses, Li *et al*. recently demonstrated the AF9 YEATS domain to (1) span N-terminal residues 1–138, (2) form an immunoglobulin fold family β-sandwich with eight antiparallel β strands separated by nine loops capped by α helices and (3) function as a reader protein, recognizing and binding H3K9ac (ref. 19). Given their reported homology^19^, we used the crystallographic three-dimensional structure of the AF9 YEATS domain as a template to construct a computational model for MLLT1 YEATS domain. The MLLT1 YEATS sequence is 82% identical to AF9 and has no amino acid insertions or deletions (Fig. 1a). Therefore the two structures are expected to be extremely similar, resulting in a highly reliable computational three-dimensional model for the MLLT1 YEATS domain, with a QMEAN (qualitative model energy analysis) *Z* score of +0.49. (The QMEAN *Z* score ranges from −4 (worse) to +4 (best) with the average QMEAN *Z* score for high-resolution X-ray structures being 0) (ref. 20). The AF9 amino acids demonstrated by Li *et al*. to bind H3K9ac are located in loops 1, 4 and 6; these amino acids are identical in MLLT1 (Fig. 4a,b). Important residue differences between MLLT1 and AF9 involve positions 107 and 111 within loop 8 (L8) and these are the same amino acids demonstrated by Li *et al*. to be responsible for binding of AF9 to the −5 position with respect to the H3K9ac motif (H3K4). It is striking that all *MLLT1* mutations specifically affect L8; mutations involving positions 111 and 112 directly impact L8, and those involving position 117 insert NHL or the similar NHH into the β8 strand (both sequences are equally capable of binding with the adjacent β1 strand in the place of the native NHL), pushing the native NHL out of β8 and into L8 (Fig. 4a–c). Our computational homology modelling indicates that all the mutations could be accommodated within the overall YEATS fold without significantly distorting it (Fig. 4d). Indeed, when we produced wild-type and mutant MLLT1 recombinant polypeptides, the mutations did not significantly affect the protein's heat stability measured by differential scanning fluorimetry (melting temperature with 95% confidence interval for the mean of three experiments: wild-type *T*m=47.3±0.3 °C, p.117_118insNHL *T*m=47.1±0.1 °C, p.111_113NPP>K *T*m=47.3±0.3 °C, p.112_114PPV>L *T*m=46.7±0.3 °C), arguing against loss of function through simple YEATS domain misfolding. (We did not test p.117_118insHHL, because this mutant was predicted to show the same structure and hence thermostability and binding characteristics as p.117_118insNHL).

The AF9 YEATS domain has its highest affinity to H3K9ac, with somewhat lower affinity binding to H3K27 (ref. 19). We therefore tested the ability of recombinant wild-type and mutant MLLT1 YEATS domain polypeptides to bind acetylated and non-acetylated H3K9 and H3K27 using isothermal titration calorimetry (ITC). Wild-type MLLT1 bound to H3K9ac with a dissociation constant *K*_d_ of 60 μM, but failed to bind to non-acetylated H3K9 (Fig. 4e and Supplementary Fig. 1). The p.117_118insNHL mutation abolished the interaction with H3K9ac, whereas p.112_114PPV>L weakened it (*K*_d_=107 μM). The p.111_113NPP>K mutation did not affect the binding affinity with H3K9ac (*K*_d_=57 μM), but altered the changes in entropy and enthalpy upon association, suggesting that the atomic interactions and dynamics differ from wildtype. With regard to binding to H3K27ac, wild-type MLLT1 showed substantially reduced affinity compared with H3K9ac (*K*_d_=140 μM,) and did not bind non-acetylated H3K27. The p.111_113NPP>K mutant polypeptide bound H3K27ac with an even lower affinity (330 μM) (Supplementary Fig. 1). The p.112_114PPV>L and p117_118insNHL mutants showed no detectable binding to H3K27ac under the conditions used. Thus, the novel *MLLT1* mutations we describe have a clear effect on the capacity of MLLT1 YEATS to interact with H3 tail peptides. Compared with MLLT1, the AF9 YEATS domain bound H3K9ac and H3K27 with a higher affinity (*K*_d_ of 3.7 and 7.0 μM, respectively)^19^. This suggests the possibility that MLLT1 may require additional or different contacts for high-affinity binding than the ones tested.

## Discussion

MLLT1 is a critical component of the super-elongation complex (SEC) that regulates mRNA elongation by RNA polymerase II (RNAPII) during transcription, and hence regulates RNA expression^21^,^22^,^23^. Perturbation of transcriptional elongation has been shown to result in abnormal development and neoplastic transformation (reviewed in ref. 24). The SEC contains a number of proteins, including AFF4, MLLT1 and PTFEb, among others. Mechanistically, the C-terminal region of MLLT1 (or the homologous AF9) binds to the scaffolding protein AFF4, which then binds the positive transcription elongation factor b (PTEFb) and ELL2 through separate domains. The N-terminal YEATS domain binds the polymerase-associated factor complex (PAFc), which in turn associates with RNAPII and the chromatin template. PTEFb functions to phosphorylate the carboxy-terminal domain of RNAPII to facilitate transcriptional elongation^25^. MLLT1 and AF9 therefore bridge PTEFb (C terminus) with PAFc (N terminus) and thereby with RNAPII, enabling transcriptional elongation. MLLT1 and AF9 compete with each other for binding AFF4 at their C terminus and exist in separate SEC complexes^23^; however, the functional differences between MLLT1- and AF9- bearing SEC complexes are not clear. Support for a critical role for MLLT1 within cellular biochemistry is provided by genetic ablation experiments in which MLLT1-deficient embryos die extremely early in utero, even before implantation^26^.

The *MLLT1* and *AF9* genes were originally recognized through their participation in chromosomal translocations that fuse their C-terminal domains (eliminating their YEATS domain) in-frame with the N terminus of MLL (eliminating the histone H3K4 methyltransferase activity but retaining the chromatin template binding function) within acute leukaemia^27^,^28^. These translocations result in bridging of PTEFb with RNAII similar to that described above, yet with important differences. The N terminus of MLL (which itself is able to bind PAFc and to the chromatin template of MLL-specific targets) fuses with MLLT1 (or AF9), thereby again bridging RNAPII with SEC and enabling the transcription of MLL target genes such as *MEIS* and *HOXA9* (refs 21, 22, 23, 29). It has long been speculated that the YEATS domain likewise functions by binding the chromatin template^30^,^31^; if this is the case, the specific genetic targets of MLLT1 likely differ from those of MLL.

In addition to AFF4 of the SEC, the C terminus of both MLLT1 and AF9 also bind to DOT1L^19^,^32^,^33^,^34^, which is the sole enzyme responsible for H3K79 methylation^35^,^36^. DOT1L was originally thought to be part of the SEC complex, however, evidence now suggests that the interaction between MLLT1/AF9 and AFF4 competes with the binding of MLLT1/AF9 with DOT1L (refs 22, 23). Li *et al*. recently demonstrated that the AF9 N-terminal YEATS domain specifically binds H3K9ac, bridging the chromatin template with DOT1L, resulting in H3K79 trimethylation, and hence transcriptional activation of associated genes^19^. They thereby establish that the AF9 YEATS domain serves as an acetyl-lysine reader protein and suggest that it functions similarly with respect to SEC (ref. 19). The >80% homology between AF9 and MLLT1 YEATS domain supports a similar function for MLLT1. MLLT1 has been previously demonstrated to bind to histones 1 and 3, however the preferred moiety for binding has not yet been identified^30^.

In the current study, we identified recurrent *MLLT1* mutations that all target L8 of the YEATS domain. We demonstrated that wild-type MLLT1 indeed binds H3K9ac and that the *MLLT1* mutations result in proteins with altered binding to H3K9ac. Therefore, our binding assays confirm that L8 plays an important role in modulating the affinity and specificity of the YEATS domain, and document a functional impact of the recurrent *MLLT1* mutations. However, even wild-type MLLT1 binds to H3K9ac with a much lower affinity than does AF9. Therefore, it remains possible, if not likely, that H3K9ac may not be the histone acetyl-lysine that MLLT1 binds to with the highest affinity. The underlying complexity of the functional impact of *MLLT1* mutation is also suggested by the fact that reduced binding of AF9 with H3K9ac resulted in decreased gene expression^19^; the increased expression of critical genes that we report and the presumed gain of function resulting from *MLLT1* mutation are therefore difficult to explain. One of many possibilities is that decreased binding of mutant-MLLT1-bearing SEC may result in increased binding of AF9-bearing-SEC (which has a higher H3K9ac binding affinity than MLLT1). Further functional studies are needed to fully explore MLLT1 histone tail binding specificity. Moreover, the alteration in histone binding may not fully account for the functional impact of these mutations.

Other than histones, the only other protein known to bind the N terminus of MLLT1 is PAF1, a member of the PAF multi-protein complex that binds to the SEC and is also involved in cell cycle regulation, miRNA processing, H3K4 methylation and H3K79 methylation^37^,^38^. The dysregulation of several *HOX* genes and the upregulation of *MYC* (*MYC-C*) in MLLT1-mutant FHWT provide indirect support for an altered interaction between PAFc and the SEC in MLLT1-mutant tumours, as these genes are all recognized to be regulated through transcriptional elongation via the SEC (refs 23, 39, 40, 41). The positive effect of the *MLLT1* mutations on *MYC* expression may contribute to transformation through activation. The PAF complex is composed of four proteins, PAF1, CTR9, CDC73 and LEO1. Of considerable interest is the recent report of germline inactivating mutations within the *CTR9* gene in three of 35 Wilms tumour families^42^,^43^, and the report of the development of Wilms tumours in patients with germline inactivating CDC73 mutations (hyperthyroidism-jaw tumour syndrome, OMIM 145001) (refs 44, 45). These were coupled with somatic mutations of the remaining alleles, consistent with tumour suppressor function. Both CTR9 and CDC73 play a negative role in transcriptional elongation^45^, therefore inactivating mutations in these genes and activating mutations of *MLLT1* may similarly result in transcriptional activation. We did not find somatic mutations in any of the genes in the PAFc in these 77 patients. Functional studies will be of considerable interest to define the precise changes in binding between mutant versus wild-type MLLT1 and components of the PAFc in addition to histones.

The absence of reports of these novel *MLLT1* mutations in other tumours or tissues suggests the possibility that their impact may be restricted to a specific cellular context. Further, the observation that these mutations were identified in very young patients whose tumours arose within ILNR, precursor lesions thought to develop quite early in renal development^2^,^46^, provides evidence that this particular cellular context may be limited to the early embryonic kidney. Many WT with WT1 mutations also present at a young age, occur within ILNRs and develop as early as the intermediate mesoderm^5^. Constitutional *WT1* mutations often result in multiple ILNRs and WTs^46^. These *WT1*-mutant tumours of infancy require Wnt pathway activating mutations (for example, *CTNNB1*, *WTX*) for transformation^5^,^47^. It is therefore noteworthy that 37% of the evaluable MLLT1-mutant tumours (which lacked *WT1* mutations and deletions) also had *CTNNB1* or *WTX* mutations, and *MLLT1*-mutant tumours all show a striking increase in expression of *PITX2* (Fig. 2), a gene encoding a homeobox protein whose expression follows canonical Wnt pathway activation and results in increased proliferation during early development^48^,^49^. The identification of multiple ILNRs in some patients with MLLT1-mutant WTs is intriguing, and raises questions regarding the possibility of germline mutations. We were unable to identify any evidence of *MLLT1* germline mutations, even in the MLLT1-mutant tumours with multiple ILNRs. Nor were we able to identify *MLLT1* mutations in normal kidney adjacent to MLLT1-mutant tumours. Last, one of the authors (VH) provided DNA samples from 48 families with familial WT. These were tested for *MLLT1* mutations and all were negative (data not shown). In summary, we did not find evidence to support the existence of germline *MLLT1* mutations in FHWT. It may be speculated that in patients with ILNRs and whose tumours carried *MLLT1* mutations, these mutations occurred in an undifferentiated cell early in kidney development. The resulting clone of undifferentiated cells could remain localized, or as the early kidney grows they could separate into multiple aggregates, resulting in the subsequent development of single or multiple ILNRs. WT may then develop within an ILNR, possibly following mutations resulting in Wnt pathway activation. This model would predict that, unlike the adjacent normal kidney tissue we assessed for several patients with MLLT1-mutant tumours, ILNRs from these patients would carry the *MLLT1* mutation. Unfortunately, ILNR material was unavailable to test this.

Evidence for HOX dysregulation mediated by *MLLT1* mutation in FHWT is most abundantly seen in the striking and early overexpression of *HOXA13*. *HOXA13* is not expressed in normal renal development, and the specific role of *HOXA13* overexpression in WT development is not apparent. However, it is critical for the development of the distal limbs and the reproductive system; in fact, *HOXA13* mutation is responsible for the rare hand-foot-uterus syndrome (OMIM 140000). *HOXA13* upregulation has also been shown to be a marker of poor outcome in a number of adult cancers (reviewed in ref. 50). Mechanistically, the association between *MLLT1* mutation and *HOXA13* overexpression seen in our mutant transfection analysis is supported by the accompanying increased expression of *HOTTIP*, a lncRNA that has been reported to result in transcriptional activation of *HOXA13* (ref. 12). The aberrant expression of critical lncRNAs (and their targets) in MLLT1-mutant FHWT may simply reflect underlying general mechanisms affecting both lncRNAs and targets. However, one might speculate that MLLT1 may participate more broadly in the regulation of transcription mediated by lncRNAs, in keeping with a proposed model whereby lncRNAs act as scaffolds that brings regulatory protein complexes (such as SEC) together into larger functional units in order to coordinate cell-type-specific gene expression programmes^51^.

In summary, we report novel somatic mutations in *MLLT1* and provide evidence that supports a role for *MLLT1* mutation in the aetiology of a subset of FHWT. Our data indicates that understanding the function of these *MLLT1* mutations could provide important insight into the function of the YEATS domain in other proteins, and into transcriptional regulation during early renal development as well as tumour development.

## Methods

### TARGET data management

The NCI TARGET initiative specifies and supports the long-term deposition and maintenance of the data files, methods and quality control steps involved in the comprehensive genomic analysis of TARGET samples. The sequencing BAM files from both WES and WGS are deposited in the Sequence Read Archive at the National Center for Biotechnology Information, and are accessible through the database of genotypes and phenotypes (dbGAP, http://www.ncbi.nlm.nih.gov/gap) under the accession number phs000471. Gene expression, chromosome segmental copy number and the clinical information are available through the TARGET data matrix (http://target.nci.nih.gov/dataMatrix/TARGET_DataMatrix.html). These are annotated within MIAME compliant MAGE-TAB files fully describing the methods, the specimen processing details and the quality control parameters.

### Specimens

Primary, pre-therapy tumour and paired blood or normal kidney samples from 77 patients registered on NWTS-5 with FHWT who subsequently relapsed were included in the discovery set. To determine the mutation frequency and to identify clinical associations, a validation set was identified comprised of a previously defined case-cohort of FHWTs likewise treated on NWTS-5 (including those in the discovery set)^5^. This approach allows for efficient evaluation of molecular markers in a tumour type characterized by a low rate of relapse. In brief, a cohort of all 1473 patients with FHWT enroled on NWTS-5 who were treated according to protocol and who had samples submitted to the NWTSG tumour bank in 2003 were identified. Following the methods of Prentice^52^, a random sample of 33% of the cohort was selected. To this group, 178 cases from the entire cohort who were known to have relapsed as of 2003 were added. This resulted in a total of 600 patients, 30% of whom relapsed. Of the 600 cases in the original case-cohort, 475 continued to have available tumour DNA samples in 2012 and received adjuvant chemotherapy. (Patients registered on the very low risk protocol who did not receive adjuvant chemotherapy were removed as all patients with *MLLT1* mutation received adjuvant chemotherapy). The validation set included tumours from these 475 patients, 139 of whom relapsed. The median time to relapse for FHWT is <1 year, and <1% of patients with FHWT relapse beyond 5 years^53^. NWTS-5 closed for accrual in 2002, therefore the clinical outcome data is fully mature. Written consent was obtained from the parents and the studies were performed with the approval of the Ann & Robert H. Lurie Children's Hospital Institutional Review Board.

### Sequencing

WGS was accomplished through the four adaptor library protocol^54^ and variants were identified through a pipeline that is described on the TARGET DataMatrix. The variants were filtered retaining all somatic, non-synonymous variants in exons with a somatic score >−10, somatic rank ⩾0.1, FET *P*<0.05. WES was performed on the Illumina HiSeq platform. Variant calling from the aligned BAM files was performed using both ATLAS and SAMtools and annotation and filtering was performed using the SACBE annotation pipeline^55^,^56^ as well as Bambino Version 1.05 (ref. 57). Pipelines for both WGS and WES identified single missense, insertion and deletion variants in exons.

### *MLLT1* variant verification

Primers were designed to amplify a 242 base-pair sequence of genomic DNA that included the sites for both the insertion and deletions identified. PCR amplification was performed using Platinum PCR SuperMix High Fidelity (Life Technologies Corporation, Carlsbad, CA). Cycling conditions were as follows: initial denaturation was performed at 96 °C for 2 min; 5 cycles were performed at 96 °C for 20 s, 60 °C for 50 s, and 72 °C for 30 s; 30 cycles were performed at 94 °C for 22 s, 55 °C for 50 s, 72 °C for 30 s; a final extension was performed at 72 °C for 10 min. The amplified product was detected using capillary electrophoresis (QIAxcel Advanced System, Qiagen, Valencia, CA). Positive samples were confirmed by Sanger sequencing using a Big Dye Terminator v1.1 cycle sequencing kit (Life Technologies Corporation, Carlsbad, CA) and M13 forward and M13 reverse primers as sequencing primers. The DNA sequence analysis was performed with 3130xl Genetic Analyzer Data Collection software v3.0 (Life Technologies Corporation, Carlsbad, CA).

Forward primer with M13F: 5′-TGTAAAACGACGGCCAGTGTGCCCTGAGAGAGAAGTGG-3′

Reverse primer with M13R: 5′-CAGGAAACAGCTATGACCTGAAGGTGAGCTTCTCG-3′

### *MLLT1* gene expression analysis

The relative expression of the mutant and wild-type alleles was determined using 2-step RT–PCR. Primers were designed to target exons 4 and 5 of the *MLLT1* gene (NM_ 005934). Complementary DNA was synthesized using the SuperScript VILO cDNA kit from Invitrogen. The manufacture protocol was followed and 300 ng of total RNA was used per reaction. Briefly, the complementary DNA (cDNA) synthesis reaction was incubated at 25 °C for 10 min, 42 °C for 120 min and 85 °C for 5 min. The PCR reaction mixture was assembled from a 2XddPCR Supermix for Probes (Bio-Rad, Cat#1863010), with a final of concentration of 900 nM for each primer and 3 ul of cDNA in a final volume of 20 μl. Cycling conditions were as follows: denaturation was performed at 95 °C for 10 min; 40 cycles were performed at 94 °C for 30 s and 59 °C for 50 s; a final extension was performed at 98 °C for 10 min. The amplified product was detected using capillary electrophoresis (3130xl Genetic Analyzer, Life Technologies Corporation, Carlsbad, CA).

*MLLT1*–RT–PCR—fwd /56-5′-FAM/CTGTTCCTGAACCTGGAAGG-3′

*MLLT1*–RT–PCR—rev 5′-TGGGTAACATGGGGTAGTCG-3′

### Chromosome segment copy number analysis

DNA labelling, hybridization, and array scanning were performed for SNP 6.0 arrays (Affymetrix, Santa Clara, CA) according to the manufacturer's protocol and processed with the Affymetrix Genotyping Console 4.0 software to generate Birdseed .chp and .txt files using the Birdseed v2 algorithm with the default parameters., Model-based expression analysis was performed and imported into R for reference normalization to a diploid reference chromosome determined for each tumour, resulting in a normalized probe level summarization value and a genotype call for each individual probe^58^. Circular binary segmentation was performed in R using the DNAcopy BioConductor package. Default parameters included nperm=10,000 (the number of permutations used for *P*value calculation), *α*=0.01 (the significance level for the test to accept change points), and random seed=12,345,678 (the seed for the random number generator). This resulted in a segmented file for each tumour sample relative to the corresponding normal sample. Segments containing at least eight markers in which the log2 value was ≥+0.5 or ≤−0.5 were considered regions of gain or loss, respectively.

### Gene expression analysis

RNA labelling, hybridization, and array scanning were performed according to the manufacturer's protocol for the Affymetrix U133 plus 2 arrays. Unsupervized analysis was performed using the Non-negative Matrix Factorization (NMF) Consensus Version 5 (ref. 59), applied to the level 3 data using the following parameters: k initial, 2; k final, 8; number of clusterings, 20; maximum number of iterations, 2,000; error function, divergence; random seed, 12,345,6789; stop convergence, 40; stop frequency, 10. Comparisons of gene expression between two groups of samples were performed using the Significance Analysis of Microarray Version 4.0 (ref. 60) with the following parameters: two-class unpaired comparison using the *T*-statistic test, 200 permutations, and a fold change cutoff of +/−2.

### Methylation analysis at 11p15

Analysis of the extent of methylation of two imprint control regions (ICR) on chromosome 11p15 was performed taking advantage of specific restriction sites recognized by methylation-sensitive enzyme as previously described^61^. In brief, DNA was extracted using the Qiagen DNeasy Blood and Tissue Kit (Qiagen, Santa Clarita, CA) following the manufacturer's protocol. DNA was digested with either *RsaI*, *RsaI* plus *HpaII* or *Rs*a*I* plus *MspI* followed by quantitative PCR. Per cent methylation was determined at each locus by comparing the quantity of amplified product after *RsaI* and *HpaII* digestion with the quantity of amplified product after cutting with *RsaI* alone. The *RsaI* and *MspI* digest was used to control for complete digestion. Retention of imprinting was defined as 30–70% methylation of both ICR1 (H19DMR) and ICR2 (KvDMR); loss of imprinting as 80–100% methylation of ICR1 and 30–70% methylation of ICR2; LOH as 80–100% methylation of ICR1 and 0–20% methylation of ICR2.

### Transfection analysis

The p117_118insNHL and the p.112_114PPV>K mutations were generated using the QuikChange II Site-Directed Mutagenesis Kit (Agilent Technologies). The wild-type gene in a pCMV6-XL5 vector (OriGene) was used as the template. This cDNA clone is not tagged and therefore contains the native stop codon. The primer pairs used to generate the appropriate mutation were obtained by using the Agilent QuikChange Primer Design software program.

Primers used to generate the c.343_351dupAACCACCTG (p.117_118insNHL) mutant: sense: 5'-CCGTGAACCACCTGAACCACCTGCGCTGCGAGAAGCT-3'; antisense: 5'-AGCTTCTCGCAGCGCAGGTGGTTCAGGTGGTTCACGG-3'. Primers used to generate the c.335_340delCGCCG (p.112_114PPV>L) mutant: Sense: 5'-CCTGGAAGGCAACCTGAACCACCTGCGC-3'; antisense: 5'-GCGCAGGTGGTTCAGGTTGCCTTCCAGG-3'.

The mutant strand synthesis reaction, the Dpn I digestion of the amplification products, and the transformation of the the XL10-Blue Gold ultracompetent cells were carried out according to the manufacturer's protocol. Briefly, 45 ul of XL10-Gold ultracompetent cells were incubated with the β-ME mix, the Dpn I digestion product was added and incubated on ice for 30 min, heat shocked at 42 °C for 30 s, and incubated on ice for 2 min. SOC broth was added and incubated at 37 °C for 1 h. Then, 100 ul of the culture was spread on a pre-warmed culture plate containing ampicillin, and incubated overnight at 37 °C. The resulting colonies were transferred to a flask containing 5 ml of LB media with ampicillin, and put on a shaker for 8 h at 37 °C. The plasmid was isolated from the cells using the Qiagen Plasmid Mini Kit, and was sequenced by using the sequencing primers provided with the Origene vector to verify the correct mutation.

HEK293 cells (purchased from American Type Culture Collection, Manassas, VA) were plated to achieve a confluency of 70–90%. The cells were transfected with the pCMV6-XL5 vector without the gene insert (control), the pCMV6-XL5 vector with the wild-type gene, the pCMV6-XL5 vector with the mutant gene, or with a 1:1 ratio of the mutant and wild-type genes by using Lipofectamine 2000 according to the manufacturer's instructions. Three independent experiments were performed for each transfection. RNA was isolated from the cells 24, and 48 and 72 h after transfection by using the Qiagen RNA Isolation Kit. Expression of the mutant was verified with the methodology described above for *MLLT1* gene expression analysis. The expression of selected genes of interest in the transfected HEK293 cells was performed using quantitative reverse transcription PCR using selected commercially available TaqMan Gene Expression Assay kits as described below.

### Western analysis

The above transfected cells were lysed in RIPA buffer at 24, 48 and 72 h after transfection, and the lysates were separated using SDS-polyacrylamide gel electrophoresis. The proteins were transferred to polyvinylidene fluoride membranes, followed by blocking with 1% bovine serum albumin in PBS with 0.05% Tween, incubation for 1 h at room temperature with a MLLT1 antibody (1:1,000, diluted in blocking buffer; HPA031166, lot#R30719, Sigma), and incubation for 30 min at room temperature with biotin-labelled secondary antibody (1:2,500 in blocking buffer; Rabbit AP, AP1000, lot#Y0222, Vector Labs). Beta-actin (primary, 1:5,000, Anti-beta-actin, A5411, lot#093K879, Sigma; secondary, 1:2,500, Mouse AP, AP2000, lot#W0426, Vector Labs) was used for the loading control under the same conditions described for MLLT1. Bands were visualized by using the BCIP/NBT Kit (SK5400, Vector Labs). See Supplementary Fig. 2 for uncropped western blot.

### Quantitative RT–PCR

Reverse transcription reactions, RNase inhibition and real-time PCR were performed according to the manufacturer's instructions for *GAPDH*, *HOXA13* and *HOTTIP*. In brief, RNA was diluted to a concentration of 5 ng μl^−1^ and the reverse transcription reactions were performed using the High Capacity cDNA Reverse Transcription Kit (Life Technologies, cat# 4368814) and RNase Inhibitor (Life Technologies, cat# 100021540), per the manufacturer's instructions. Non-amplification controls were run once for each RNA sample. Real-time PCR was performed on 3 μl of cDNA, according to the manufacturer's instructions, using the TaqMan Universal PCR Master Mix, No AmpErase UNG (Life Technologies, cat# 4324018) and the 7500 Fast Real-Time PCR System. A non-template control was run with every plate, for each gene expression assay. The following Taqman gene expression assays were used: *GAPDH* (Life Technologies, cat# Hs03929097_g1), HOXA13 (Life Technologies, cat# Hs00426284_m1) and *HOTTIP* (Life Technologies, cat# Hs00955374_s1). *GAPDH* was used as the endogenous control to calculate the dCt values for each sample. The ddCt value for the control sample at 24 h was used as the reference sample for calculating the relative quantitation (RQ) value in each sample for each gene (*RQ*=2(−ddCt)). The data are shown as the average RQ and SEM for three independent experiments.

### Computational protein structure analysis

The three-dimensional structure of residues 1–138 of wild-type MLLT1 YEATS was modelled using SwissModel^62^ based on the crystal structure of the 82% identical AF9 YEATS domain bound to H3K9ac (PDB id 4TMP). Mutant MLLT1 YEATS domains were produced using the same procedure. Models were visualized and analysed using Pymol (www.pymol.org).

### Recombinant protein analysis

The codon-optimized DNA sequence coding for residues 1–138 of human MLLT1 (established using the Gibson assembly method) was inserted into the pET28 vector with thrombin cleavage site between 6His tag and protein sequence. Proteins were expressed in *Escherichia coli* BL21(pLysS) cells. Expression was induced using 1 mM IPTG, and allowed to proceed for 8 h at 20 °C. Wild-type and mutant proteins were purified using Ni-NTA sepharose followed by size exclusion chromatography (Pharmacia Superdex 75 16/600). Peak fractions were pooled. For ITC analysis proteins were dialyzed into 50 mM Hepes buffer pH 7.4, 300 mM NaCl, 5 mM β-mercaptoethanol and placed in the measurement cell (YEATS domain) or the injection syringe (histone peptides) of MicroCal ITC200 and TA Nano microcalorimeters, at 25 °C. Protein concentrations for wild-type, p.117_118insNHL, p.112_114PPV>L and p.111_113NPP>K mutants were 120, 130, 120 and 90 μM, respectively. Peptide concentrations for all measurements were 1.2 mM. Thermal stability of wild-type and mutant proteins was probed using a Roche LightCycler 480II. Wild-type and mutant proteins were kept in the ITC buffer at concentrations of 7 μM. SyproOrange was used as dye, and temperature was ramped from 20–95 °C. Reported values are mean values of triplicate measurements.

### Sequencing of selected genes

Probes to *CTNNB1*, *WT1* and *WTX* were designed using RefSeq IDs limited to coding exons and UTRs with probe density specified at 2x with 98.7% of the target region covered. Pooled libraries were hybridized to the RNA probes. Post-capture material was purified and enriched with 10 cycles of PCR. Paired-end 100 base reads were sequenced per pool in a single lane of an Illumina HiSeq2500 instrument. Single nucleotide variants were filtered out if they were judged not to be damaging by at least 2/3 of the following algorithms: SIFT using Ensembl Variant Effect Predictor Version 75 (ref. 63), PolyPhen Version 2 (ref. 10) or Provean Version 1.1.3 (ref. 64).

## Additional information

**Accession codes:** Whole genome and whole exome sequencing data have been deposited in the dbGAP database under the accession code phs000471.

**How to cite this article:** Perlman, E. J. *et al*. *MLLT1* YEATS domain mutations in clinically distinctive Favourable Histology wilms tumours. *Nat*. *Commun*. 6:10013 doi: 10.1038/ncomms10013 (2015).

## Supplementary Material

Supplementary Information Supplementary Figures 1-2 and Supplementary Tables 1-2

## Figures and Tables

**Table 1:** Features of FHWT with *MLLT1* mutations.

**Age (months)**	**Relapse**	**Histology**	**Stage**	**Nephrogenic Rests**	**11p15 Status**	**Protein**
22	Relapse	Mixed	II	None	ROI	p.117_118insNHL
5	Relapse	Mixed	II	ILNR	ROI	p.117_118insNHL
10	No	Blastemal	II	None	ROI	p.117_118insNHL
7	No	Mixed	I	None	ROI	p.117_118insNHL
24	No	Blastemal	III	None	LOH	p.117_118insNHL
10	Relapse	Mixed	III	ILNR	ROI	p.117_118insNHL
6	No	Mixed	II	multiple ILNR	ROI	p.117_118insNHL
1	No	Mixed	II	uncertain	ROI	p.117_118insNHL
27	Relapse	Mixed	III	None	ROI	p.117_118insNHL
50	Relapse	Mixed	III/IV	‘possible'	ROI	p.117_118insNHL
5	Relapse	Mixed	II	None	ROI	p.117_118insNHL
9	No	Mixed	I	ILNR	ROI	p.117_118insNHL
17	No	Mixed	I	multiple ILNR	ROI	p.117_118insNHL
12	Relapse	Mixed	III	ILNR	ROI	p.117_118insHHL
1	No	Mixed	II	multiple ILNR	ROI	p.117_118insHHL
21	No	Mixed	I	ILNR	ROI	p.117_118insHHL
20	No	Mixed	II	multiple ILNR	ROI	p.111_113NPP>T
26	Relapse	Mixed	III	ILNR	ROI	p.111_113NPP>K
77	Relapse	Mixed	III	None	LOI	p.112_114PPV>L

ILNR, Intralobar nephrogenic rest; LOH, loss of heterozygosity; LOI, Loss of imprinting; ROI, retention of normal imprinting.
