# Supplementary material for: MLLT1 YEATS domain mutations in clinically distinctive Favourable Histology Wilms tumours
Source: Nat Commun. 2015 Dec 4;6:10013. doi: 10.1038/ncomms10013 (PMC4686660; doi:10.1038/ncomms10013)
Supplement: Supplementary Information — Supplementary Figures 1-2 and Supplementary Tables 1-2 [file ncomms10013-s1.pdf]

Supplementary Figure 1.

A.

H3K9ac vs H3K9

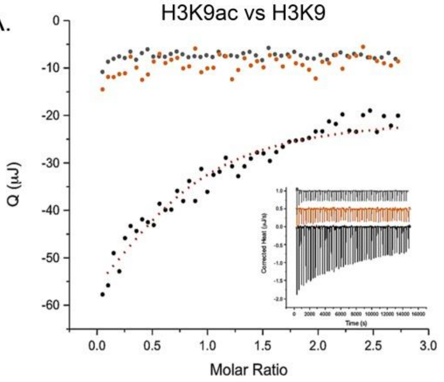

	wt_h3k9ac
Ka (1/M)	1.68E+04
ΔH (kJ/mol)	-10.26
n	0.69
Kd (μM)	59.69
ΔS (J/mol·K)	46.44
ΔG (kJ/mol.K)	-24.10

B.

Wild-type: H3K9ac vs H3K27ac

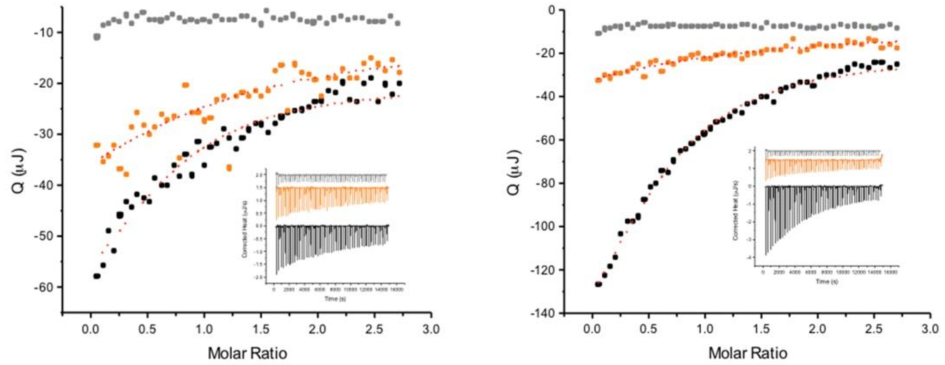

	wt_h3k9ac	m2_h3k9ac	wt_h3k27ac	m2_h3k27ac
Ka (1/M)	1.68E+04	1.75E+04	7.05E+03	3.04E+03
ΔH (kJ/mol)	-10.26	-32.12	-8.252	-16.36
n	0.69	0.629	1.08	0.872
Kd (μM)	59.69	57.11	141.80	329.10
ΔS (J/mol·K)	46.44	-26.49	45.99	11.79
ΔG (kJ/mol.K)	-24.10	-24.23	-21.96	-19.87

Binding characteristics of recombinant wild-type and mutant MLLT1 YEATS domain polypeptides to H3 tails

A. The interaction between MLLT1 YEATS and H3K9 depends on K9 acetylation.
ITC data comparing the reaction of MLLT1 YEATS (in the measurement cell) with H3K9ac (black), H3K9 (orange), and buffer control (gray). Integrated heats are shown. The inlay shows raw heats after baseline subtraction, offset for better visibility.

B. MLLT1 YEATS discriminates between H3K9ac and H3K27ac.
ITC data comparing the reaction of MLLT1 YEATS (wild-type and NPP>K mutant) with H3K9ac (black), H3K27ac (orange),and buffer control (gray). Integrated heats are shown. The inlay shows raw heats after baseline subtraction, offset for better visibility.

Supplementary Figure 2.

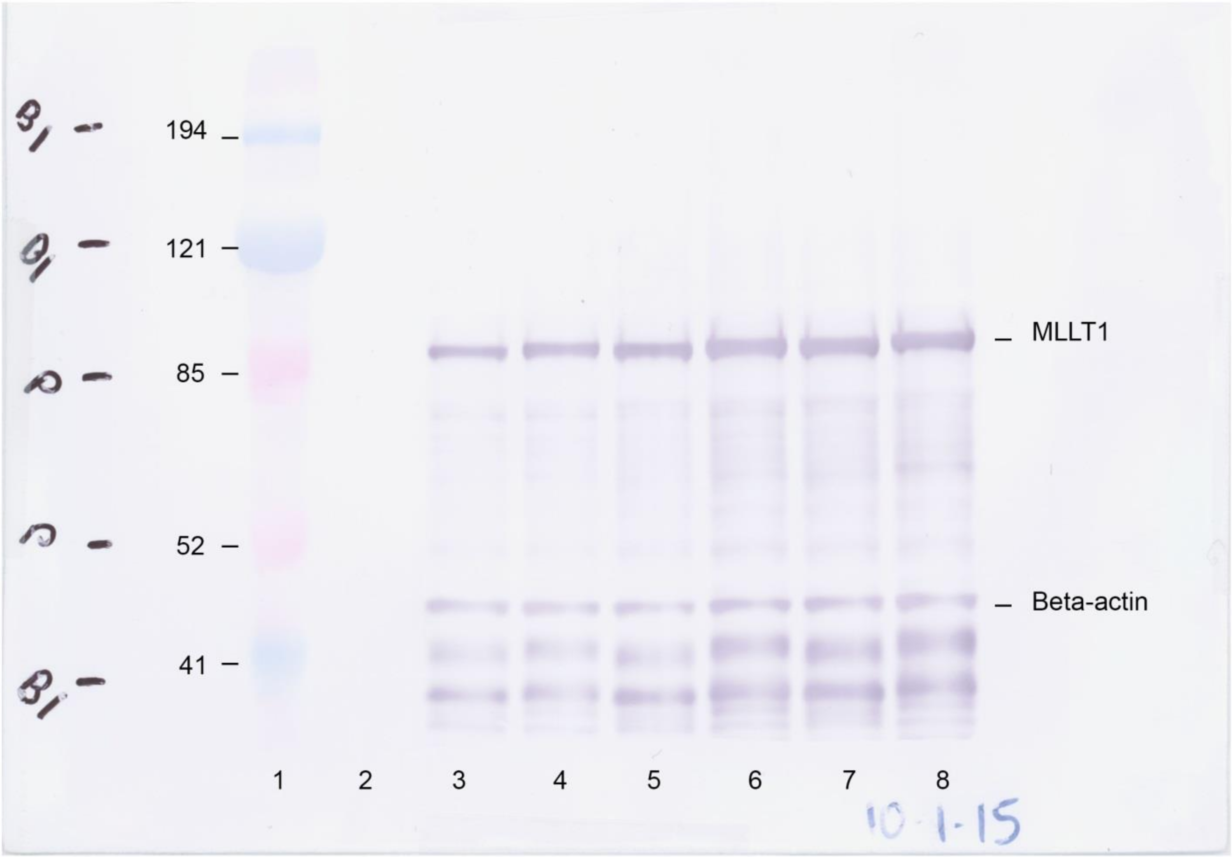

MLLT1 and Beta-actin western blot in HEK293 cells transfected with wild-type and mutant MLLT1

HEK293 cells were transfected with wild-type or p.117_118insNHL mutant MLLT1 and protein was isolated at 24, 48, and 72 hours. The lanes contain the following: 1, marker; 2, empty; 3, wild-type MLLT1 at 24 hours; 4, p.117_118insNHL mutant MLLT1 at 24 hours; 5, wild-type MLLT1 at 48 hours; 6, p.117_118insNHL mutant MLLT1 at 48 hours; 7, wild-type MLLT1 at 72 hours; 8, p.117_118insNHL mutant MLLT1 at 72 hours.

Supplementary Table 1: Details of mutations and copy losses identified in 77 FHWT illustrated in Figure 2

TARGET_CASE_ID	Gene Symbol	Variant Classification	Allelic Fraction	Reference Allele	TumorSeq Allele1	TumorSeq Allele2	Chromosome	Start position	End position	Transcript Strand	Transcript Position	Protein Change	Number of markers	Segment mean (log2)
	MLLT1	INSERT	0.68			CAGGTGGTT	19	6230649	6230649	-	515_516	p.117_118insNHL		
PAJMUF	CTNNB1	MISSENSE	0.35	A	A	G	3	41266124	41266124	+	277	p.T41A		
PAJNSL	MLLT1	INSERT	0.49			CAGGTGGTT	19	6230649	6230649	-	515_516	p.117_118insNHL		
PAKSCC	MLLT1	INSERT	0.7			CAGGTGGTT	19	6230649	6230649	-	515_516	p.117_118insNHL		
PALERC	MLLT1	INSERT	0.73			CAGGTGGTT	19	6230649	6230649	-	515_516	p.N115_L117dup		
	MLLT1	DELETE+	0.52	CGGGCGGG	CGGGCGGG	CT	19	6230661	6230668	-	497_504	p.111_113NPP>K		
PAECJB	CTNNB1	MISSENSE	0.47	C	C	T	3	41266137	41266137	+	290	p.S45F		
PAJNDU	MLLT1	DELETE	0.5	CGGGCG	CGGGCG		19	6230661	6230666	-	499_504	p.112_114PPV>L		
PAJNLT	MLLT1	INSERT	NA			CAGGTGGTT	19	6230649	6230649	-	515_516	p.117_118insNHL		
PAJNEC	DGCR8	MISSENSE	0.89	G	G	A	22	20079439	20079439	+	1902	p.E518K		
	DGCR8	MISSENSE	0.90	G	G	A	22	20079439	20079439	+	1902	p.E518K		
PAJMIZ	SIX1	MISSENSE	0.38	**T**	**T**	**C**	14	61115378	61115378	-	778	p.Q177R		
	DGCR8	MISSENSE	0.96	G	A	A	22	20079439	20079439	+	1902	p.E518K		
	WT1	COPY LOSS					11	198,510	48,705,843				33790	-1.43
PAKZFK	WTX	COPY LOSS					X	63,154,896	63,985,808				349	-1.29
	DROSHA	MISSENSE	0.31	C	C	T	5	31421465	31421465	-	3798	p.E1147K		
CAAAAL	SIX1	MISSENSE	0.53	**T**	**T**	**C**	14	61115378	61115378	-	778	p.Q177R		
	DROSHA	MISSENSE	0.57	C	C	T	5	31421465	31421465	-	3798	p.E1147K		
CAAAAO	SIX2	MISSENSE	0.45	T	T	C	2	45235720	45235720	-	823	p.Q177R		
	DROSHA	MISSENSE	0.46	C	C	T	5	31421465	31421465	-	3798	p.E1147K		
	SIX2	MISSENSE	0.33	T	T	C	2	45235720	45235720	-	823	p.Q177R		
PAJMSE	WT1	COPY LOSS					11	28,158,725	134,944,770				67780	-1.96
	DROSHA	MISSENSE	0.56	C	C	T	5	31421465	31421465	-	3798	p.E1147K		
PALDTE	SIX2	MISSENSE	0.56	T	T	C	2	45235720	45235720	-	823	p.Q177R		
	DROSHA	MISSENSE	0.48	T	T	C	5	31421452	31421452	-	3811	p.D1151G		
PAJMEL	CTNNB1	MISSENSE	0.96	C	A	A	3	41266125	41266125	+	278	p.T41N		
PALKCW	DROSHA	MISSENSE	0.49	T	T	G	5	31421452	31421452	-	3811	p.D1151A		
	DROSHA	NONSENSE	0.41	G	G	A	5	31515145	31515145	-	1599	p.R414*		
PAKZHF	DROSHA	NONSENSE	0.78	G	G	A	5	31526904	31526904	-	495	p.Q46*		
PAJMFY	SIX1	MISSENSE	0.37	**T**	**T**	**C**	14	61115378	61115378	-	778	p.Q177R		
PAKRVH	SIX1	MISSENSE	0.41	**T**	**T**	**C**	14	61115378	61115378	-	778	p.Q177R		
PAJPAU	SIX2	MISSENSE	0.85	T	T	C	2	45235720	45235720	-	823	p.Q177R		

	CTNNB1	DELETE	0.3	CTT	CTT		3	41266134	41266136	+	287_289	p.S45del		
PAJLNJ	WT1	NONSENSE	0.93	T	A	A	11	32450079	32450079	-	929	p.K245*		
PAKKSE	CTNNB1	MISSENSE	0.43	A	A	C	3	41266110	41266110	+	263	p.H36P		
	WTX	NONSENSE	1.00	T	A		X	63412098	63412098	-	1342	p.K357*		
PAJPEW	WT1	COPY LOSS					11	27,708,726	32,810,106				3234	-1.6
PAKGZX	WTX	FRAMESHIFT	0.80	-	-	G	X	63412684	63412685	-	755_756	p.S161fs		
PAKYFC	WTX	NONSENSE	0.58	G	G	A	X	63412095	63412095	-	1345	p.R358*		
PALDWP	WTX	NONSENSE	0.89	G	A		X	63412095	63412095	-	1345	p.R358*		
PAEAFB	WTX	NONSENSE	0.87	G	G	A	X	63412095	63412095	-	1345	p.R358*		
PAJLUJ	WT1	NONSENSE	0.54	G	G	T	11	32417910	32417910	-	1338	p.S381*		
	WT1	FRAMESHIFT	0.95		G	G	11	32417924	32417924	-	1323_1324	p.P376fs		
PAJNUS	WTX	COPY LOSS					X	63,077,515	64,635,785				652	-2.61
CAAAAC	WTX	COPY LOSS					X	15,714,942	155,233,846				75730	-1.51
	WT1	COPY LOSS					11	18,970,736	67,906,985				28550	-1.33
CAAAAS	WTX	COPY LOSS					X	168,465	155,233,846				85897	-1.83
PAJLSP	WTX	COPY LOSS					X	63,389,353	63,919,767				231	-0.66
	WT1	COPY LOSS					11	32,340,278	32,458,219				162	-4.12
PAJMJT	WTX	COPY LOSS					X	58,082,334	102,782,426				20834	-2.4
PAJMKJ	WT1	COPY LOSS					11	10,609,248	134,944,770				81176	-1.46
PAJNNC	WTX	COPY LOSS					X	58,549,556	100,317,090				19494	-0.5
PAJPDC	WTX	COPY LOSS					X	47,486,445	68,409,345				7707	-2.17
PAJPGY	WTX	COPY LOSS					X	63,378,320	63,460,890				33	-1.85
PAKULH	WT1	COPY LOSS					11	4,976,999	48,660,461				31228	-2.36
PALJIP	WT1	COPY LOSS					11	18,971,163	114,541,075				59681	-1.44
PAJNCZ	WTX	COPY LOSS					X	63,032,314	64,542,241				629	-1.91
PALGAZ	WT1	COPY LOSS					11	11,562,006	121,057,430				69604	-0.57
PAJLWT	WTX	COPY LOSS					X	63,225,253	63,512,160				111	-2.53
PAJPHA	WTX	COPY LOSS					X	63,211,128	63,494,265				108	-1.64
	WT1	COPY LOSS					11	32,196,933	32,412,622				193	-1.42
PAJNZU	WTX	COPY LOSS					X	63,211,128	63,488,591				104	-1.66
PAKFME	WTX	COPY LOSS					X	63,161,742	63,461,588				117	-2.83
PAKNTW	WTX	COPY LOSS					X	63,395,315	64,162,392				363	-0.77
PAKZER	WTX	COPY LOSS					X	63,351,704	63,460,890				43	-2.98
PALGLU	WTX	COPY LOSS					X	63,284,786	63,412,151				54	-2.44
	WT1	COPY LOSS					11	198,510	82,809,600				50709	-0.88
PAKMUB	WTX	COPY LOSS					X	63,154,896	63,730,533				226	-1.83
PALKRS	WTX	COPY LOSS					X	61,919,000	68,677,000				3378	-1.01
PAJNAV	WT1	COPY LOSS					11	25,722,066	50,520,259				16200	-2.23
	WT1	COPY LOSS					11	198,510	55,363,328				35011	-2.22
PAJNCC	WTX	COPY LOSS					X	58,549,556	100,317,090				19494	-0.5

Supplementary Table 2: Genes Differentially Expressed in *MLLT1* mutant FHWT using SAM analysis (q-value <0.05).

Gene ID	Fold Change	p value
HOXA13	127.68	1.9E-06
PRAC	107.94	1.3E-07
PITX2	46.50	2.2E-05
MCTP2	25.50	1.4E-06
DNER	15.76	1.0E-07
LY6G6D	12.33	7.1E-07
MYC	10.58	1.7E-04
OLFM3	9.28	1.2E-04
ABCB1	9.21	2.3E-02
TNRC9	9.06	1.7E-05
IRX5	9.05	2.2E-03
PRAC2	8.73	1.4E-04
LOC644695	8.40	1.9E-03
IRX3	8.21	1.1E-11
C2ORF40	7.82	9.7E-04
CLDN11	7.69	1.9E-03
SUSD5	7.48	3.4E-03
KIAA1679	6.86	5.0E-03
TRIM43	6.62	1.4E-02
LOC196541	5.42	1.8E-02
CXCL11	5.33	4.1E-02
KCND2	5.21	1.0E-02
ABCB1	5.03	4.1E-03
SLC27A6	4.99	5.6E-03
PCDH20	4.76	2.7E-03
C6ORF155	4.69	1.3E-05
HNT	4.47	2.4E-21
OGFRL1	4.38	3.8E-05
PPAPDC1A	4.35	4.1E-02
LOC374491	4.34	2.9E-03
NEGR1	4.25	2.1E-02
SPTLC2L	4.18	5.5E-03
CPNE4	4.17	4.3E-02
VEGFC	4.04	1.3E-02
LHCGR	4.02	4.7E-02
RASSF6	4.01	1.6E-02
F12	4.01	8.9E-07
TTC29	3.80	9.5E-03
GCOM1	3.78	1.8E-03
GPR39	3.67	7.8E-03
NFKBIZ	3.45	4.6E-04
RELN	3.29	4.5E-04
FZD10	3.16	1.2E-03
RFXDC1	2.91	2.4E-02
KCNIP4	2.82	3.6E-02
SNAI2	2.68	1.4E-03
HS3ST5	2.61	1.1E-02
C4ORF19	2.54	1.0E-02

CTH	2.30	2.4E-03
SAMD13	2.30	1.6E-02
ACSBG1	2.15	6.0E-03
LOC389073	2.14	3.8E-02
DMRTB1	2.10	4.0E-02
RP11-484I6.3	2.07	3.0E-02
ADAMTSL1	2.07	6.3E-03
ADAMTS20	2.06	2.0E-03
ACSL3	0.46	2.3E-04
SLC37A1	0.46	3.2E-03
KCNH2	0.44	9.6E-04
ZC3HAV1	0.44	3.0E-08
NUP210	0.43	3.6E-03
SNX9	0.43	1.6E-05
LOC91461	0.42	8.8E-03
TAGLN2	0.40	1.6E-03
S100A11	0.40	2.4E-04
FOXP1	0.40	6.3E-03
F11R	0.39	2.6E-02
CARD10	0.38	7.9E-04
FLJ22184	0.38	5.4E-05
MGAT4A	0.37	7.0E-06
ADA	0.37	5.4E-05
HOXD9	0.37	2.4E-02
PHLDB3	0.35	2.1E-03
SEMA6A	0.35	2.6E-03
PRSS8	0.34	1.2E-03
FGF8	0.33	7.5E-03
MFNG	0.33	2.5E-05
SIX1	0.32	4.3E-03
KBTBD11	0.32	7.4E-03
ZBTB46	0.32	4.8E-03
STAMBPL1	0.29	1.9E-04
HOXD8	0.29	2.2E-02
MXRA8	0.28	1.7E-04
GRTP1	0.27	5.2E-03
IGF2	0.26	4.8E-02
GLT25D2	0.24	2.3E-05
UGT3A1	0.23	2.2E-04
MMP23	0.21	7.6E-05
LOC375295	0.16	5.2E-05
PABPC5	0.13	1.7E-27
HTR2B	0.08	3.6E-04
HOXD10	0.08	1.9E-02
HOXD11	0.08	5.8E-03
LOC440173	0.08	1.8E-02
SCG5	0.04	1.5E-05
